# Interventions for primary (intrinsic) tracheomalacia in children

**DOI:** 10.1590/S1516-31802013000100013

**Published:** 2013-02-01

**Authors:** Vikas Goyal, Brent I. Masters, Anne B. Chang

## Abstract

**BACKGROUND:**

Tracheomalacia, a disorder of the large airways where the trachea is deformed or malformed during respiration, is commonly seen in tertiary paediatric practice. It is associated with a wide spectrum of respiratory symptoms from life-threatening recurrent apnoea to common respiratory symptoms such as chronic cough and wheeze. Current practice following diagnosis of tracheomalacia includes medical approaches aimed at reducing associated symptoms of tracheomalacia, ventilation modalities of continuous positive airway pressure (CPAP) and bi-level positive airway pressure (BiPAP), and surgical approaches aimed at improving the calibre of the airway (airway stenting, aortopexy, tracheopexy).

**OBJECTIVES:**

To evaluate the efficacy of medical and surgical therapies for children with intrinsic (primary) tracheomalacia.

**METHODS:**

*Search*: The Cochrane Airways Group searched the Cochrane Central Register of Controlled Trials (CENTRAL), the Cochrane Airways Group’s Specialized Register, Medline and Embase databases. The Cochrane Airways Group performed the latest searches in March 2012.

*Selection criteria:* All randomized controlled trials (RCTs) of therapies related to symptoms associated with primary or intrinsic tracheomalacia.

*Data collection and analysis:* Two reviewers extracted data from the included study independently and resolved disagreements by consensus.

**MAIN RESULTS:**

We included one RCT that compared nebulized recombinant human deoxyribonuclease (rhDNase) with placebo in 40 children with airway malacia and a respiratory tract infection. We assessed it to be a RCT with overall low risk of bias. Data analyzed in this review showed that there was no significant difference between groups for the primary outcome of proportion cough-free at two weeks (odds ratio (OR) 1.38; 95% confidence interval (CI) 0.37 to 5.14). However, the mean change in night time cough diary scores significantly favoured the placebo group (mean difference (MD) 1.00; 95% CI 0.17 to 1.83, P = 0.02). The mean change in daytime cough diary scores from baseline was also better in the placebo group compared to those on nebulized rhDNase, but the difference between groups was not statistically significant (MD 0.70; 95% CI -0.19 to 1.59). Other outcomes (dyspnoea, and difficulty in expectorating sputum scores, and lung function tests at two weeks also favoured placebo over nebulized rhDNase but did not reach levels of significance.

**AUTHORS’ CONCLUSIONS:**

There is currently an absence of evidence to support any of the therapies currently utilised for management of intrinsic tracheomalacia. It remains inconclusive whether the use of nebulized rhDNase in children with airway malacia and a respiratory tract infection worsens recovery. It is unlikely that any RCT on surgically based management will ever be available for children with severe life-threatening illness associated with tracheomalacia. For those with less severe disease, RCTs on interventions such as antibiotics and chest physiotherapy are clearly needed. Outcomes of these RCTs should include measurements of the trachea and physiological outcomes in addition to clinical outcomes.

**This is the abstract of a Cochrane Review published in the Cochrane Database of Systematic Reviews (CDSR) 2012, issue 10, art. No. CD005304. DOI:** 10.1002/14651858.CD005304.pub (http://onlinelibrary.wiley.com/doi/10.1002/14651858.CD005304.pub3/abstract?systemMessage=Wiley+Online+Library+will+be+disrupted+on+17+December+from+at+23%3A00+GMT+%2818%3A00+EST%29+for+essential+maintenance+for+approximately+one+hour). For full citation and authors details see reference 1.

**The full text is freely available, for Latin America and the Caribbean, from:** http://cochrane.bvsalud.org/cochrane/main.php?lib=COC&searchExp=Interventions%20and%20for%20and%20primary%20and%20(intrinsic)%20and%20tracheomalacia%20and%20in%20and%20children&lang=pt (this link may be temporary)
